# In silico prediction and in vitro assessment of novel heterocyclics with antimalarial activity

**DOI:** 10.1007/s00436-023-08089-7

**Published:** 2023-12-29

**Authors:** Martha Ilce Orozco, Pedro Moreno, Miguel Guevara, Rodrigo Abonia, Jairo Quiroga, Braulio Insuasty, Mauricio Barreto, Maria Elena Burbano, Maria del Pilar Crespo-Ortiz

**Affiliations:** 1 Department of Microbiology, Universidad del Valle, Cali, Colombia; 2 Faculty of Engineering, Universidad del Valle, Cali, Colombia; 3 Department of Chemistry, Universidad del Valle, Cali, Colombia; 4 Department of Microbiology, Group of Microbiology and Infectious Diseases, Universidad del Valle, San Fernando Campus, Calle 4 B #36-00, 760043 Cali, Colombia

**Keywords:** *Plasmodium falciparum*, Heterocyclics, Antimalarial susceptibility

## Abstract

The development of new antimalarials is paramount to keep the goals on reduction of malaria cases in endemic regions. The search for quality hits has been challenging as many inhibitory molecules may not progress to the next development stage. The aim of this work was to screen an in-house library of heterocyclic compounds (HCUV) for antimalarial activity combining computational predictions and phenotypic techniques to find quality hits. The physicochemical determinants, pharmacokinetic properties (ADME), and drug-likeness of HCUV were evaluated in silico, and compounds were selected for structure-based virtual screening and in vitro analysis. Seven *Plasmodium* target proteins were selected from the DrugBank Database, and ligands and receptors were processed using UCSF Chimera and Open Babel before being subjected to docking using Autodock Vina and Autodock 4. Growth inhibition of *P. falciparum* (3D7) cultures was tested by SYBR Green assays, and toxicity was assessed using hemolytic activity tests and the *Galleria mellonella* in vivo model. From a total of 792 compounds, 341 with good ADME properties, drug-likeness, and no interference structures were subjected to in vitro analysis. Eight compounds showed IC_50_ ranging from 0.175 to 0.990 µM, and active compounds included pyridyl-diaminopyrimido-diazepines, pyridyl-N-acetyl- and pyridyl-N-phenyl-pyrazoline derivatives. The most potent compound (UV802, IC_50_ 0.178 µM) showed no toxicophoric and was predicted to interact with *P. falciparum* 1-cysperoxidredoxin (*Pf*Prx1). For the remaining 7 hits (IC_50_ < 1 μM), 3 showed in silico binding to *Pf*Prx1, one was predicted to bind the haloacid dehalogenase-like hydrolase and plasmepsin II, and one interacted with the plasmodial heat shock protein 90.

**Supplementary Information:**

The online version contains supplementary material available at 10.1007/s00436-023-08089-7.

## Introduction

Malaria is a tropical disease causing an estimated of 247 million cases and 619,000 deaths in the endemic areas as described by the World Health Organization (WHO) in 2021 (World Health Organization 2022). In the Americas, a total of 600,000 cases have been reported with the highest burden of the disease in Venezuela, Colombia, and Brazil, these countries accounting for 79% of the cases (World Health Organization 2022). From the five parasitic species affecting humans, *Plasmodium vivax* accounts for the highest morbidity, whereas *P. falciparum* is the deadliest parasite. The control of severe malaria mainly relies on the artemisinin combination therapy (ACT), which in the last decade has played a main role in reducing malaria cases and lethality in many endemic countries (World Health Organization 2022). Nevertheless, *P. falciparum* has evolved and developed resistance to artemisinin and/or their partner drugs, posing a major thread to control and achieve eradication. The development of new antimalarials is paramount to obtain malaria reduction and elimination of malaria cases in the most affected populations and particularly in pregnant women and children under 5 years old (World Health Organization 2022).

The next generation of antimalarials should provide a simple, effective, and low-cost option for therapy that may be able to overcome the parasite resistance. To achieve this, novel molecules of easy synthesis with potent and selective antimalarial activity are needed for further development. The search of active molecules has been historically performed using phenotypic approaches using whole cell-based assays; however, screening of large collections of chemical compounds is time-consuming and costly. In the last decade, in silico analysis has been incorporated to examine small molecule libraries by simulating target-ligand interactions and to infer biological activity (virtual screening); the in silico approaches also help to deduce potential targets and mechanisms of action. Diverse bioinformatic tools may predict drug-likeness and physicochemical properties to select those to be prioritized for virtual screening and further in vitro or in vivo testing. Regardless of the methodology used, the search for active molecules (hits) of quality has been challenging as many inhibitory molecules do not advance to the next steps on development. Combined strategies using in silico and phenotypic approaches may improve the detection and selection of hits likely to become leads and successfully progress through the pipeline as drug candidates.

From the diverse source of potential active molecules in the organic chemistry, heterocyclic moieties are relevant scaffolds of biological activity and therapeutic properties as many marketed drugs (> 90%) are based on heterocyclic structures. A wide range of biological activity has been mainly linked to those heterocyclics containing pyridine and dihydropyridine rings showing relevant antimicrobial, antioxidant, and anticancer effects; furthermore, chalcones and quinolines have also shown great antimalarial activity (Ramírez-Prada et al. 2017; Cuartas et al. 2020). Earlier studies by our group have revealed that 2-pyrazolines (EC_50_ 5.54 μg/mL) and thiazolyl-pyrazolines (EC_50_ ≤ 25 μM) could inhibit *P. falciparum* growth (Ramírez-Prada et al. 2017; Cuartas et al. 2020).

Given the urgent need to search for novel antimalarial compounds, the aim of this work was to screen our in-house library of heterocyclic compounds (HCUV) for antimalarial activity using computational predictions and conventional in vitro techniques to find quality hits. The physicochemical and drug-likeness properties of the HCUV library were evaluated in silico, and the active compounds were tested in vitro and in vivo for toxicity. We have identified novel compounds with antimalarial activity and their predicted targeted proteins.

## Materials and methods

**Fig. 1:**
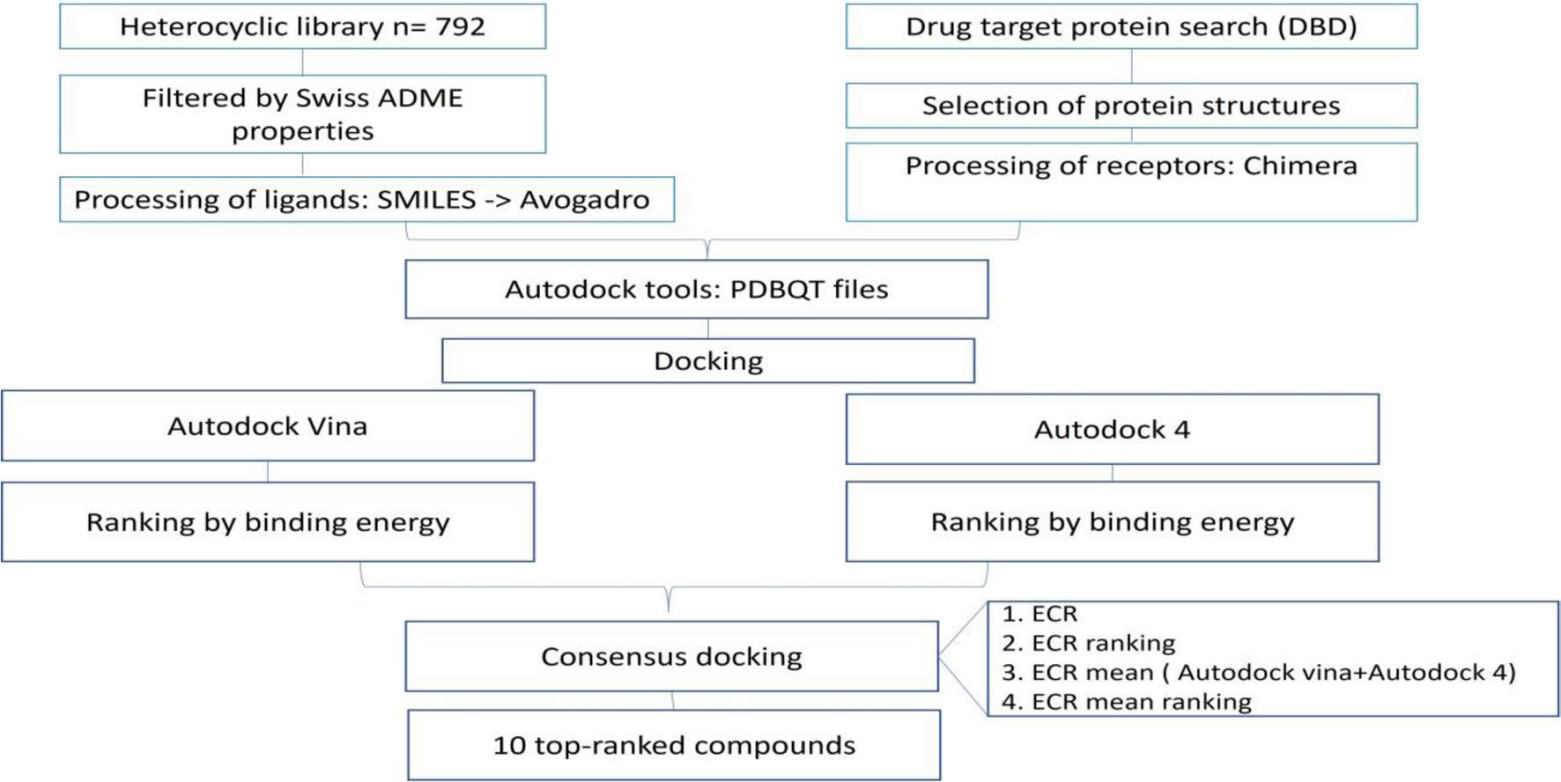
General scheme of in silico analysis for HCUV library. *ECR*, exponential consensus ranking

A collection of 792 compounds (HCUV) was synthesized at the Department of Chemistry (Universidad del Valle). The novel heterocyclics were hybrids containing ring systems or scaffolds with previously reported high biological activity. The library comprises a series of hybrids containing pyrazolines, pyrazolopyridines, pyrazolopyrimidines, pyridopyrimidines, diazepines, chalcones, quinolines, and sulfonamides. For the in silico analysis, a workflow was established as indicated in Fig. 1. Briefly, compounds were filtered by ADME properties (adsorption, distribution, metabolism, and excretion) and drug-likeness, then a structure-based analysis was performed. *Plasmodium* target proteins were selected from the DrugBank Database (DBD), and ligands and receptors were prepared and subjected to docking analysis.

### In silico physicochemical and pharmacokinetic (ADME) studies

A first filter was to select those compounds with good predicted bioavailability and pharmacological properties. The SwissADME online platform was used to examine the physicochemical properties, drug-likeness, pharmacokinetics, and medicinal chemistry of the heterocyclics (Daina et al. 2017). Physicochemical parameters evaluated were lipophilicity, size, polarity/solubility, flexibility, and saturation, whereas the pharmacokinetic parameters were gastrointestinal (GI) absorption, blood–brain barrier (BBB) permeation, glycoprotein and cytochrome interactions. Drug-likeness, a parameter used to identify features of an orally active drug, was judged by the five rule-based filters, namely, the Lipinski (Lipinski et al. 2012), Ghose (Ghose et al. 1999), Veber (Veber et al. 2002), Egan (Egan et al. 2000), and Muegge (Muegge et al. 2001) rules. Those heterocyclics with suitable chemical properties and complying, at least, the Lipinski rule of five (LRo5) were selected for in silico and in vitro screenings. Key molecular parameters in the LRo5 to keep pharmacology criteria avoiding poor absorption and permeation are based on the partition coefficient (log P < 5), molecular weight (MW < 500), hydrogen bond acceptors (HBA) and donors (HBD) < 10 and < 5, respectively, and polar surface area (PSA) < 140 Å (Lipinski et al. 2012). Medicinal chemistry parameters include lead-likeness, synthetic accessibility, and the likelihood to be false positive or pan assay interference structures (PAINS). Compounds showing inhibitory effects on parasite cultures were also subjected to in silico analysis of toxicity using ProTox-II (Banerjee et al. 2018) and radar analysis of bioavailability using SwissADME (Daina et al. 2017). ProTox-II uses several models to predict hepatotoxicity, immunotoxicity, carcinogenicity, mutagenicity, and cytotoxicity (Banerjee et al. 2018).

### Virtual screening

Docking analyses were performed to predict the potential antimalarial action of the novel compounds. A search for target proteins of *P. falciparum* was conducted in the DBD, and 3D structures were retrieved and analyzed from the Protein Data Bank (PDB, https://www.rcsb.org/ search). Fourteen protein structures with 3D crystallographic resolution between 1.8 and 2.3 Å and *R* free value < 0.26 were selected and prepared for docking (Gore et al. 2017; Bhojwani and Joshi 2019). The target proteins selected were calcium-dependent protein kinase 4, *Pf*CDPK4 (Swearingen 2012; Kumar et al. 2021); heat shock protein 90, *Pf*Hsp90 (Corbett and Berger 2010); haloacid dehalogenase-like hydrolase, *Pf*HAD1 (Park et al. 2015); 1-cysperoxiredoxin, *Pf*Prx1 (Staudacher et al. 2015); spermidine synthase, *Pf*SpdS (Burger et al. 2015; Kamil et al. 2022); purine nucleoside phosphorylase, *Pf*PNP (Madrid et al. 2008); and plasmepsin II, *Pf*PMII (Silva et al. 1996; Liu et al. 2005; Recacha et al. 2015). Structures and biology of selected proteins are shown in Table 1.

**Table 1:** Protein structures and biological characteristics

Target protein	Structure (s)	Biochemical class/function	Parasite expression/location	Effect on parasite survival
Calcium-dependent protein kinase 4 (*Pf*CDPK4)	**PDB ID: 4QOX**^**a**^	Phosphorylase/sexual replication, regulation of male gametogenesis exflagellation	All stages /cytosol and membranes	Essential for transmission (Kumar et al. 2021)
Heat shock protein 90 (*Pf*Hsp90)	**PDB ID: 3K60**	ATPase/stress response, protein folding	Endoplasmic reticulum, mitochondria, apicoplast	Developmental arrest/cell death (Dutta et al. 2022)
Haloacid dehalogenase like hydrolase (*Pf*HAD1)	PDB ID: 4QJB PDB ID: 4ZEW PDB ID: 4ZEX **PDB ID: 4ZEV**	Chaperone/isoprenoid synthesis, metabolic homeostasis	Cytosol	Essential for protein trafficking/export, secretion (Guggisberg et al. 2018)
1-cys peroxiredoxin (*Pf* Prx1)	**PDB ID: 4D73**	Oxydorreductase/cysteine-dependent antioxidant enzyme	Highly expressed in trophozoites/cytosol, apicoplast	Unknown (Kimura et al. 2013)
Spermidine synthase (*Pf*SpdS)	PDB ID: 2PTG **PDB ID: 3RIE** PDB ID: 4CWA PDB ID: 2HTE	Transferase/control and synthesis of spermidine and spermine. Growth, post-translational activation of *P. falciparum* eukaryotic translation initiation factor 5A (elF5A)	Mitochondria	Transcriptional arrest (Kamil et al. 2022)
Purine nucleoside phosphorylase (*Pf*PNP)	PDB ID: 3FOW **PDB ID: 3ENZ**	Transferase/purine synthesis	Cytosol	Impaired growth (before schizogony) (Madrid et al. 2008)
Plasmepsin-II (*Pf*PMII)	**PDB ID: 4Y6M**	Hydrolase/hemoglobin degradation	Food vacuole	Impaired hemoglobin digestion (Liu et al. 2005)

^a^Protein structures selected for docking are indicated in bold

The selected target structures were refined by removing water, ligands, and non-protein molecules using UCSF Chimera software (Butt et al. 2020). Polar hydrogen bonds were added, and non-polar hydrogen atoms were merged using Autodock tools (ADT version 1.5.6). To identify the protein druggable binding sites, the structures were uploaded to the DoGSiteScorer (Protein Plus web portal https://protein.plus) (Fährrolfes et al. 2017), and the pockets were defined by area, volume, active amino acid residues, and DrugScores (Volkamer et al. 2012). A receptor docking grid was defined by focusing the grid box on the site with a DrugScore > 0.8, and the box size was set around the active site.

#### Ligand preparation for docking

Heterocyclic structures (.sdf) were processed using Open Babel (O’Boyle et al. 2011) and Avogadro software (Hanwell et al. 2012). Three-dimensional structures were subjected to energy minimization and geometry optimization using the Merck Molecular Force Field (MMFF) method (Hanwell et al. 2012). After refinement of ligands and receptors, the.pdbqt files were generated using AutoDock Tools MGL.

#### Docking simulations

To select potential hits, the ligand-binding affinity was assessed using Autodock Vina and Autodock 4, and at least three replicates were performed. The docking protocol for each software was validated using previously known protein–ligand interactions and performing the redocking among *P. falciparum* plasmepsin I (*Pf*PMI) (PDB:1LEE) and *Pf*PMII (PDB:3QS1) and the following ligands: androstan-17-one, ethyl-3-hydroxy-(5-alpha)-torreyol, delta-cadinene, alpha-cadinol, neoclovenoxid, guaicoal, and artemisinin (Fatimawali et al. 2021). Predicted binding energies obtained using our docking protocol were similar to those redocking scores previously reported, for example, the binding energy variation for *Pf*PMII ranged from − 0.8 to 0.8 (SD 0.596), whereas for *Pf*PMI ranged from − 0.2 to 0.2 (SD 0.310). Artenimol (PubChem CID 6918483) also known as dihydroartemisinin (DHA), the active metabolite of artemisinin, was included as a control for docking simulations with all protein structures. Binding energy values were used to choose the best receptor structure when more than one structure from each protein was available in PDB (Bhojwani and Joshi 2019). A consensus docking method based on the ranking of binding energy values was used to improve the docking analysis and predictions as stated by Triches et al. (2022). Briefly, the average of the binding energy scores obtained from Autodock Vina and Autodock 4 were ranked; for Autodock 4, the binding poses based on the root-mean-square deviation (RMSD) were considered for analysis. An exponential consensus ranking (ECR) was calculated, and ECR values for the compounds with each protein structure obtained using both software tools were combined and ranked again to identify the top 10 ligands (Triches et al. 2022). The best 10 molecules from the final ranking were selected.

#### Protein–ligand interactions

For the most potent compounds in vitro, the LigPlot software (Wallace et al. 1995) was used to examine the molecular interactions with a role in inhibition. This software provides a 2-dimensional representation of intermolecular interactions and their strengths, indicating hydrogen bonds and hydrophobic contacts.

### In vitro antiplasmodial activity of heterocyclic compounds

Antimalarial activity of the selected compounds was assessed by growth inhibition assays. *P. falciparum* 3D7 parasites (a chloroquine-sensitive strain) kindly supplied by the Biodefense and Emerging Infections Research Resources Repository (BEI resources, NIAID, NIH, USA) were cultured by the Trager-Jensen method (Trager and Jensen 1976; Moll et al. 2013) with modifications. Briefly, parasite-infected red blood cells (RBCs) were kept in continuous culture in complete culture medium (CCM) containing RPMI 1640 (GIBCO), 25 mM hydroxypipazine-N’-2-ethane sulfonic acid (HEPES, Sigma), and 25 μg/mL gentamicin. The medium was further supplemented with 0.21 mM hypoxanthine (Sigma) and 10% pooled human serum. Cultures were incubated at 37 °C under a controlled atmosphere (5% CO_2_, 1% O_2_, and 94% N_2_) (Moll et al. 2013). Parasite growth was monitored by preparing Giemsa-stained smears. The parasitemia was determined by examination of at least 10 high power magnification fields (hpf) (Moll et al. 2013), and levels of parasitemia were kept between 5 and 10%.

#### Drug solutions

Artemisinin (Sigma) and chloroquine diphosphate salt (Sigma) were used as control drugs. Primary stocks of artemisinin (Art 0.16 mM) and chloroquine (CQ 0.16 mM) were prepared in dimethyl sulfoxide (DMSO) and water, respectively. Test compounds in solid powder were dissolved in DMSO at 100 mg/mL concentrated stock solutions, then secondary stock solutions (100 μM) were made in incomplete culture medium iCCM (i.e., RPMI 1640 containing HEPES and gentamicin) which were generally diluted 1:10 into the final suspensions.

Susceptibility assays were performed by the SYBR Green method with modifications (Kato et al. 2018). Briefly, *P. falciparum*-infected RBCs cultured under standard conditions were plated at about 2% parasitemia (2% hematocrit), in 96-well microplates, and 10 μM of each compound was added; the final DMSO concentration was less than 0.1%. Parasites were initially incubated for 72 h before addition of lysis buffer (20mM Tris-HCl, 5mM EDTA, 0.008% saponin, 0.08% Triton X-100) supplemented with 0.04% SYBR™ Green I Nucleic Acid (10,000 × in DMSO, Invitrogen), then the microplates were returned to culture for 4 h at room temperature and read in a microplate fluorimeter (Cytation 3 M, Biotek) at excitation 485 nm–emission 530 nm. A control drug for total growth inhibition, a non-drug well and uninfected RBCs were included as positive and negative controls, respectively. The percentage (%) of inhibition was calculated as follows: 1-(fluorescence of treated/nontreated cultures) × 100. Compounds showing at least 50% inhibition at the screening concentration (10 μM) were further tested to determine the concentration required to produce 50% inhibition of growth (IC_50_). To do this, parasite cultures were exposed to serial two-fold dilutions of the test compounds ranging from 10 to 0.078 μM. Controls of uninfected RBCs and CQ-treated infected RBCs were included, and the assays were performed in triplicate. The dose response-inhibition analysis and IC_50_ calculations were done using GraphPad Prism 9.5.1 by applying the inhibitor vs response parameters.

### Toxicity assays

#### Hemolytic activity

The ability to induce membrane damage was assessed by testing the hemolytic activity of those compounds showing inhibition of parasite growth. The assay was adapted from Sousa et al. (2021) with modifications. Briefly, 180 μL of human RBCs adjusted to 2% hematocrit in phosphate buffered saline (PBS) were placed into a 96-well microplate and subsequently exposed to 200 μM of each selected compound. As a positive control, 20 μL of 1% sodium dodecyl sulfate (SDS) was added, and a non-drug well was included as a negative control. Free hemoglobin was measured after 72 h of incubation at 37 °C by spectrophotometry at 540 nm (Cytation 3 M, Biotek). Nonspecific absorbance was corrected by subtracting the blank (PBS). Determinations were done by triplicate in at least two independent experiments. Compounds with hemolytic activity at 200 μM were further tested at 100 μM, 50 μM, and 25 μM dilutions.

### In vivo toxicity assays

In vivo toxicity was performed using the invertebrate *Galleria mellonella* model. Healthy, beige larvae weighing 200–250 mg were obtained from the Entomology laboratory at the University and selected for toxicity assays. Groups of ten six-instar larvae were inoculated with 10 μL or 20 μL of each compound at concentrations equivalent to 3000 mg/kg and incubated at 37 °C in darkness. The larval survival was monitored every 24 h for 5 days to determine the half-lethal dose (LD_50_). The larvae were initially injected with 1000 mg/kg, and if most larvae (> 60%) survived after 5 days, the assay was performed using higher doses up to 3000 mg/kg (Piatek et al. 2021).

## Results

### Filtering of compounds by ADME and drug-likeness properties

From the total of 792 compounds, 364 (46%) showed good membrane permeability and oral/gastrointestinal bioavailability and complied with the LRo5. Only 17 (2.2%) compounds of the total collection were detected as PAINs structures. A total of 357 compounds with no Lipinski violations and suitable physicochemical properties were subjected to further in silico analysis. From those, 236 (66.1%) compounds showed no violations for Ghose, Veber, Egan, and Muegge rules.

### In silico screening for antimalarial prediction

**Fig. 2:**
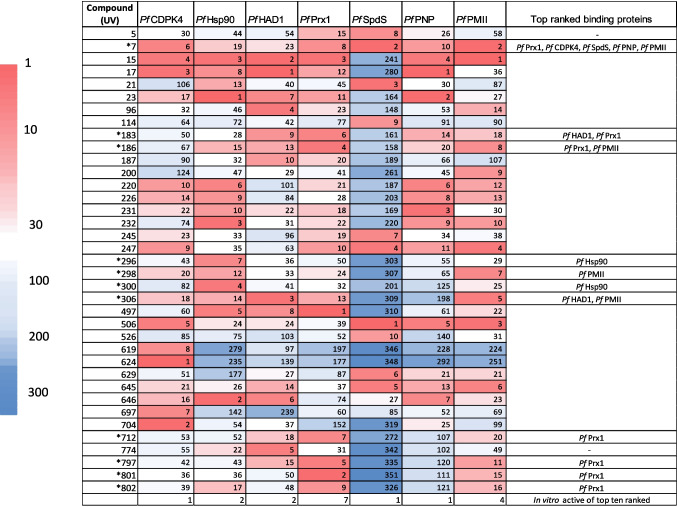
In silico top ranked heterocyclic compounds. Heatmap showing the compounds in the top 10 ranking with at least one target protein (*n* = 37). The ranking is represented by the figures in the cells. Asterisks indicate those compounds with in vitro antimalarial activity; the predicted binding proteins for these compounds are shown in the last column of the heatmap. The last row of the heatmap indicates the number of top binders for each protein showing in vitro activity

Seven critical proteins from *P. falciparum* were selected and screened as drug targets in this study. After filtering, the HCUV library was docked with one structure from each target protein; the best structures were selected based on performance (Bhojwani and Joshi 2019) as follows: *Pf*CDPK4 PDB ID: 4QOX, *Pf*Hsp90 PDB ID: 3K60, *Pf*HAD1 PDB ID: 4ZEV, *Pf*Prx1 PDB ID: 4D73, *Pf*SpdS PDB ID: 3RIE, *Pf*PNP PDB ID: 3ENZ, and *Pf*PMII PDB ID: 4Y6M (Table 1). The average of the binding energy values for each protein using Autodock Vina and Autodock 4 were ranked before consensus docking was performed and top-ranked ligands were identified as shown in Fig. 2. In general, the correlation of Autodock Vina and Autodock 4 predictions was variable and depending on target, as judged by scatter plots of the rankings from each software showing better fit for *Pf*Hsp90 (*r*^2^ 0.540), *Pf*CDPK4 (*r*^2^ 0.519), *Pf*SpdS (*r*^2^ 0.519), *Pf*PMII (*r*^2^ 0.507), and *Pf*Prx1 (*r*^2^ 0.494). Little or poor correlation was seen with *Pf*HAD1 (*r*^2^ 0.3645) and *Pf*PNP (*r*^2^ 0.0231) (Supplementary information S1).

Thirty-seven compounds (37/357, 10.4%) were predicted to have relevant binding (top 10 ranking) for at least one of the docked target proteins (Fig. 2). Twenty-nine compounds were chalcone derivatives (mainly quinoline and pyrazoline hybrids), 4 diazepines, 3 indazoles, and one sulfonyl-acetophenone (a precursor molecule for chalcone synthesis) (Supplementary information S2). Compound UV15 ((quinolinyl)amino-N-acetyl-pyrazoline) and compound UV7 ((quinolinyl)amino-chalcone) were top ranked for predicted binding with 6 and 5 out of the 7 *Plasmodium* target proteins, respectively. Other two compounds, UV17 ((quinolinyl)amino-N-acetyl-pyrazoline) and UV247 ((quinolinyl)amino-chalcone) and UV506 (a triazinyl-amino chalcone), showed the best ranking with 4 proteins. Four compounds (4/37, 10.8%) were top ranked for binding with 3 proteins, 7 (7/37, 18.9%) with 2 proteins, and the remaining 20 (20/37, 54%) were predicted to bind one target protein (Supplementary information S2). Nevertheless, not all the predicted binders showed in vitro antiplasmodial activity (Fig. 2).

### In vitro analysis

**Fig. 3:**
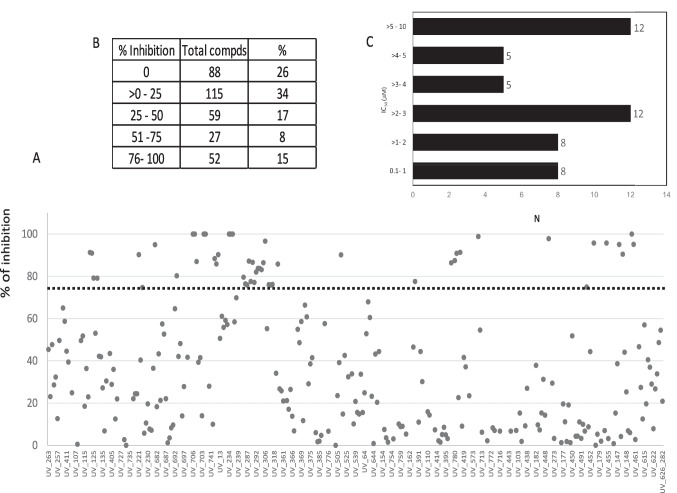
In vitro antimalarial activity of the HCUV library. **A** Inhibition (%) of *P. falciparum* cultures. Dotted line indicates 75% inhibition. **B** Distribution of compounds by level of inhibition. **C** IC_50_ of reproducible active compounds (*n* = 50)

A total of 341 compounds were tested for growth inhibition of 3D7 *P. falciparum* cultures, 203 (60%) were little or no active (< 30% inhibition), and 79 (23.2%) were active showing inhibition of > 50% at 10 μM (Fig. 3A, B). The IC_50_ was performed in compounds with inhibition > 50% as follows: 50 compounds (14.7%) had IC_50_ < 8 μM in a reproducible manner and from those 8 compounds (16%) had IC_50_ ranging from 0.178 to 0.991 μM (Fig. 3C).

The most potent compounds (8 compounds with IC_50_ < 1 μM) had 4-pyridylamino substitutions and belonged to four series as follows: pyridyl-diaminopyrimido-diazepines UV797, UV801, UV802; pyridyl-amino-N-acetyl pyrazolines UV286, UV292; pyridyl-amino-N-phenyl pyrazolines UV 296, 297; and one pyridyl-amino-N-4-chlorophenyl pyrazoline UV306. Other 8 compounds (16%) showed IC_50_ ranging from 1 to 2 μM; compounds showing IC_50_ slightly higher than 1 μM were the 4-(quinolinyl)amino chalcone UV7 and the pyridyl-aminopyrimido-diazepine UV712. The remaining compounds showed IC_50_ ranging from 2 to 5 μM (22, 44%) and > 5–8 μM (12, 24%). Eight compounds with IC_50_ < 1 μM were considered hits, and the pyridyl-aminopyrimido-diazepines UV801 and UV797 were the most inhibitory compounds (Table 2).

**Table 2:**
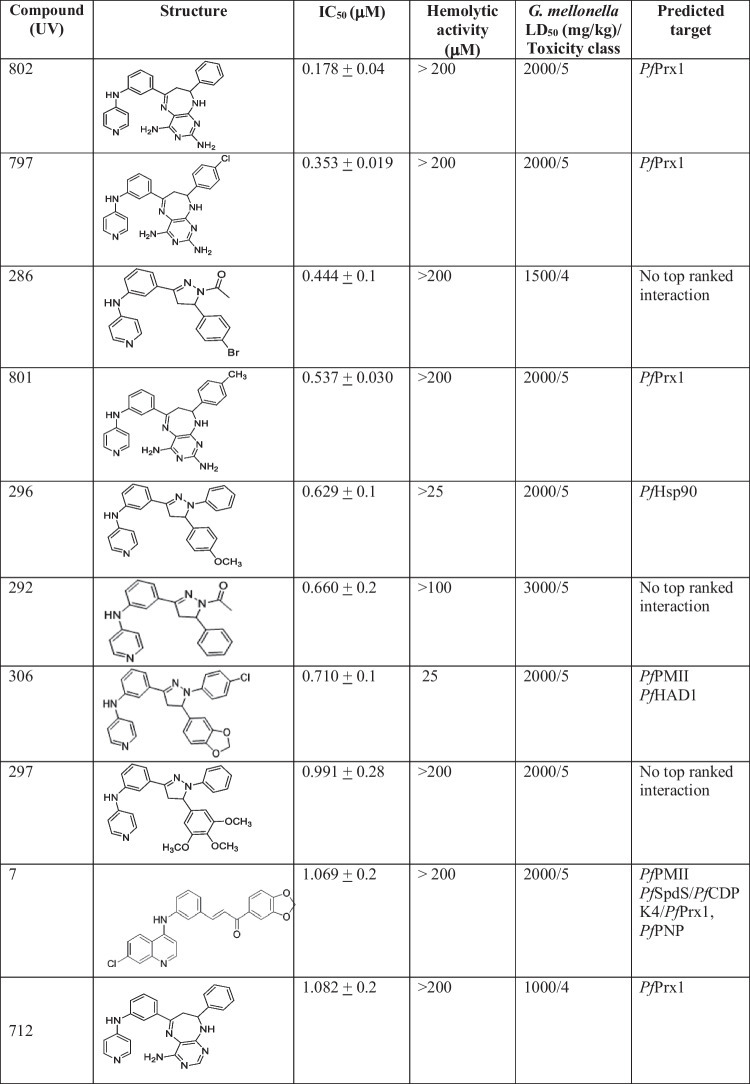
Structure, toxicity, and predicted targets of the most active compounds

Data from in vitro assays and in silico predicted targets are depicted in this table

**Fig. 4:**
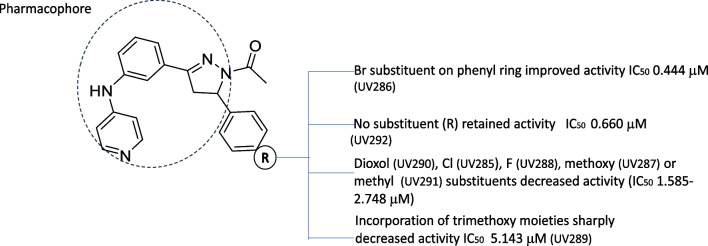
SAR analysis of N- acetyl pyrazoline series

The qualitative structure activity relationship (SAR) analysis revealed that for the 4-(pyridyl-aminophenyl)-diaminopyrimido-diazepine, compound UV802 showed the most potent inhibitory effect (IC_50_ 0.178 μM), and chloro (UV797) or methyl (UV801) substitutions on phenyl group of pyrimido-diazepine system decreased activity (Table 2). The pyridylamino substituted acetyl-diphenyl-pyrazoline derivatives having an additional coordinating bromide atom on phenyl group, UV286, had the highest potency (IC_50_ 0.444 μM), and elimination (UV292) or substitution of the bromide atom decreased the antimalarial activity (UV290, UV285, UV288, UV287, UV291, UV289) (Fig. 4). N-diphenyl-pyrazoline derivatives with a pyridylamino substitution also inhibited *P. falciparum* cultures, showing that the methoxy-phenyl derivative UV296 was the most active (IC_50_ 0.629 μM); by contrast, further addition of methoxy groups resulted in decreased antimalarial activity (UV 297 IC_50_ 0.991 μM) (Table 2).

### In silico vs in vitro correlation

From the total of 37 compounds predicted to have binding affinity with the selected target proteins, 11 showed in vitro parasite growth inhibition (IC_50_ 0.178–2.4 μM, Fig. 2), whereas from the 10 most active compounds in vitro, 7 were top ranked with at least one of the selected target proteins (Table 2). Compounds UV 801, 802, and 797 having the most potent activity (IC_50_ < 1 μM) were top ranked with *Pf*Prx1; these compounds belonged to the same series of diaminopyrimido-diazepines. Compound UV296 (4-(pyridyl-aminophenyl)-methoxyphenyl-*N*-phenyl pyrazoline) was top ranked with *Pf*Hsp90, and compound UV306 (4-(pyridyl-aminophenyl)-methyledioxyphenyl-*N*-(4-chlorophenyl)pyrazoline) was top ranked with *Pf*HAD1 and *Pf*PMII. Other compounds with in vitro activity (IC_50_ ~ 1 μM) and good ranking were UV7 4-(quinolinyl)-amino chalcone) which was multitargeted to 5 proteins; compound UV712 (aminopyrimido-phenyl diazepine) was ranked for *Pf*Prx1. From the compounds showing growth inhibition lower than 1 μM, three (UV 286, UV292, and UV297) were not identified as top binders of any of the selected target proteins, probably suggesting a different target (Table 2). The remaining compounds showed lower activity in vitro (IC_50_ > 1μM); compound UV298 ((4-(pyridyl-aminophenyl)-methyledioxyphenyl-*N*-phenyl pyrazoline) was top ranked to *Pf*PMII, and compound UV300 (4-(pyridyl-aminophenyl)-N-phenyl pyrazoline) was top ranked for *Pf*Hsp90. Two compounds, UV183 and UV186, from the series of quinazoline derivatives with discrete inhibition (2.15–2.38 μM, respectively) showed interaction with *Pf*Prx1 and *Pf*HAD1 and with *Pf*Prx1 and *Pf*PMII, respectively (Table 2).

When analyzing the predictions by target, it was found that 7 out of 10 (top ranked) with predicted binding to *Pf*Prx1 showed inhibition of parasite cultures, whereas 4 out of 10 (top ranked) of those predicted to interact with *Pf*PMII showed *P. falciparum* growth inhibition; the other targets showed less agreement between in vitro activity and in silico predictions. This suggests a better correlation of in silico-in vitro assays for *Pf*Prx1 and *Pf*PMII than for the other selected targets (Table 2).

**Fig. 5:**
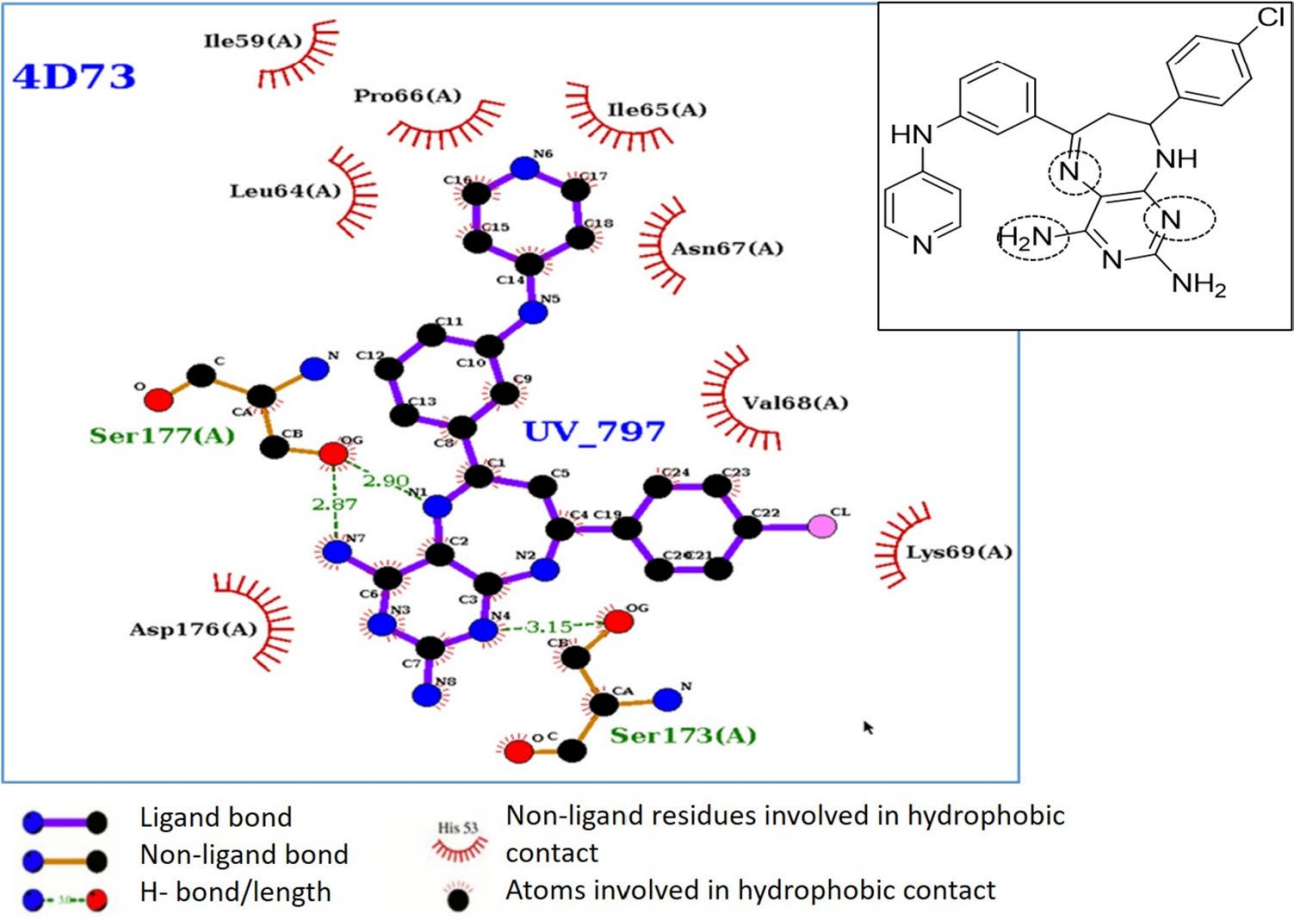
Molecular interactions between the docked complex UV797:*Pf*Prx1. Interactions of one representative diaminopyrimido-diazepine (UV797) and *Pf*Prx1 are shown. H bonds and the length are shown, and hydrophobic contacts are also indicated. The inset highlights nitrogen atoms of the pyrimido-diazepine system and the amino group of pyrimidine nucleus involved in H bonding

Analysis of protein–ligand interactions of the most active compounds, UV802 and UV797, showed that Ser 173 and Ser 177 forms contacts through hydrogen bonds (Fig. 5). Furthermore, hydrophobic interactions occur between the ligand and the residues Ile59, Leu64, Ile65, Pro66 ASn67, Val68, Lys69, and Asp176. This matches the hydrophobic interactions observed with the control ligand (artenimol: CID 6918483) hydrogen bonds: Leu64, Asn67 and hydrophobic contacts: Ile 59, Ile65, Pro66, Val68, His96).

### ADMETox-analysis of active compounds

**Fig. 6:**
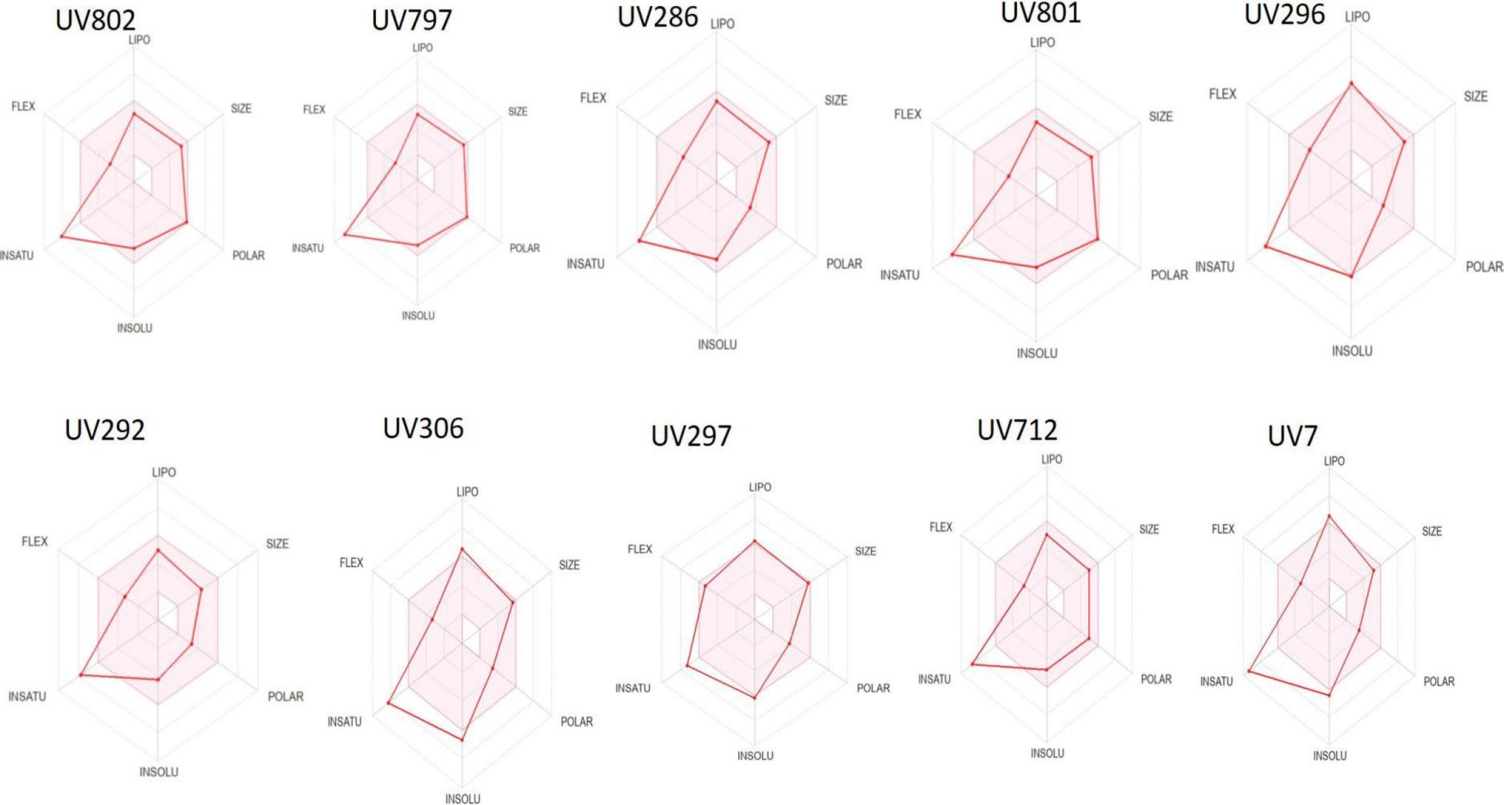
Bioavailability radars of active compounds. The radar area indicates the optimal range for solubility (INSOL), saturation (INSATU), lipophilicity (LIPO), polarity (POLAR), and molecular weight (SIZE)

Oral bioavailability radars were built for the most active compounds (IC_50_ ≤ 1μM), using SwissADME. This analysis describes the drug-likeness of a molecule using 6 properties of lipophilicity, size, polarity, insolubility, saturation, and flexibility (Fig. 6). For each of these parameters, the radar represents the physicochemical space within the best range of lipophilicity (XLOGP3 –0.7 and + 5.0), molecular weight (150–500 g/mol), polarity (Topological PSA, 20–130 Å, solubility (log S − 6 and 0), saturation (≥ 0.25), and flexibility (≤ 9 rotatable bond) (Daina et al. 2017). Three compounds showed deviations on the lipophilicity values (UV296, UV306, and UV7), whereas all 10 compounds showed a deviation in saturation; this deviation was lower in compound UV297 but still outside the physicochemical space. Compounds should comply with all 6 properties to be considered orally active. It was observed that physicochemical parameters of the most active compounds seemed to have lower values of lipophilicity and solubility than those for inactive compounds except for polarity (PSA) and molecular refractivity (MR) values; however, this trend was not significant.

The computed ADME, physicochemical properties, drug-likeness, and medicinal chemistry predictions are summarized in Table 3.

**Table 3:** Physicochemical properties, ADME, and medicinal chemistry predictions of active compounds (IC_50_ ≤ 1 μM)

Compound (UV)	802	797	286	801	296	292	306	297	7	712	Expected value
MW	436.51	456.93	435.32	436.51	420.51	356.42	468.93	480.56	428.87	407.47	< 500
PSA	127.13	127.13	57.59	127.13	49.75	57.59	58.98	68.21	60.45	101.11	< 140
HBA	4	4	3	4	3	3	4	5	4	4	< 10
HBD	4	4	1	4	1	1	1	1	1	3	< 5
RB	4	4	5	4	6	5	5	8	5	4	< 10
Log P	2.92	3.11	3.82	2.92	4.57	3.21	4.92	4.52	5.04	3.08	0–5
Log S (ESOL)	− 4.91	− 5.20	− 5.12	− 4.91	− 6.05	− 4.21	− 6.68	− 6.19	− 6.44	− 4.75	> − 6
Ghose	1	1	0	1	1	0	1	2	1	1	^a^
Veber	0	0	0	0	0	0	0	0	0	0	^b^
Egan	0	0	0	0	0	0	0	0	1	0	^c^
Muegge	0	0	0	0	1	0	1	1	1	0	^d^
GI absorption	High	High	High	High	High	High	High	High	High	High	-
BBB permeation	No	No	Yes	No	Yes	Yes	Yes	No	No	No	-
Pgp substrate	Yes	Yes	No	Yes	Yes	Yes	Yes	Yes	No	Yes	-
CYP1A2^e^ inhibitor	No	No	Yes	No	Yes	Yes	Yes	No	Yes	No	-
CYP2C19 ^e^ inhibitor	Yes	Yes	Yes	Yes	Yes	Yes	Yes	Yes	Yes	Yes	-
CYP2C9 ^e^ inhibitor	No	No	Yes	No	Yes	Yes	Yes	Yes	Yes	No	-
CYP2D6 ^e^ inhibitor	Yes	Yes	Yes	Yes	Yes	Yes	Yes	Yes	No	Yes	-
CYP3A4^e^ inhibitor	Yes	Yes	Yes	Yes	Yes	Yes	Yes	Yes	Yes	Yes	-
Synthetic accessibility	4.46	4.33	3.70	4.46	3.97	3.65	4.10	4.37	3.26	4.23	< 6
Bioavailability score	0.55	0.55	0.55	0.55	0.55	0.55	0.55	0.55	0.55	0.55	> 0.1

^a^MW: 160–460, LogP: − 0.4–5.6, HBA 20–70, molar refractivity MR: 40–130

^b^PSA ≤ 140, rotable bounds RB ≤ 10

^c^ LogP: < 5.88 PSA: < 131.6

^d^ MW: 200–600, LogP − 2–5, HBA ≤ 10, HBD ≤ 5, MR ≤ 15, PSA ≤ 150, #Ring ≤ 7, #Carbons > 4, Heteroatoms > 1

Number of violations for Ghose, Veber, Egan, and Muegge rules is shown. All compounds complied with LRo5

^e^ CYP isoforms of CYP450, *Pgp* P-glycoprotein

According to the results, all compounds showed high GI absorption; UV286, UV292, UV296, and UV306 showed permeation in the BBB. Eight out of 10 compounds interacted with P-glycoprotein (Pgp) a transmembrane drug transporter. Most compounds interact with the cytochrome isoforms, indicating potential drug interactions and biotransformation mediated by the interacting cytochrome, UV286 has BBB penetration and no interaction with Pgp. Compounds UV286 and UV292 showed no violations in all 5 rule filters: Lipinski, Ghose, Veber, Egan, and Muegge. Most compounds showed no violations for Veber and Egan rules, whereas 4 compounds had violations of Muegge, and most compounds (8/10) showed violations of Ghose. For the medicinal chemistry parameters, the bioavailability score for all compounds suggests > 10% bioavailability in rats or Caco cells permeability (Martin 2005). Compounds were of easy synthesis as judged by the synthetic accessibility index ranging from 3.26 to 4.46 (Ertl and Schuffenhauer 2009). Compound UV7 reported one predicted BRENK alert.

Toxicity, median lethal dose (LD_50_), and toxicity classification were predicted for the 10 most active compounds. Eight compounds (8/10) showed predicted hepatotoxicity, 3/10 immunotoxicity, 6/10 carcinogenicity, and 4/10 mutagenesis. None of the compounds were associated to cytotoxicity. Toxicity predictions revealed low or no toxicity alerts for the pyridyl-diaminopyrimido-diazepines (UV802, UV797, and UV801) and compound UV306, whereas for compounds UV286 and UV292 showed alerts for hepatotoxicity and carcinogenicity. Pyridyl-N-phenyl pyrazolines UV 296 and 297 and the quinolinyl-amino chalcone UV7 reported at least three toxicity alerts (hepatotoxicity, carcinogenicity, and mutagenesis); however, in most cases, the predicted likelihood of toxicity was low (consensus score: ≤ 0.7) (Table 4).

**Table 4:** Protox II toxicity predictions of the most active compounds (IC _50_ ≤ 1 μM)

Compound (UV)	802	797	286	801	296	292	306	297	7	712
Hepatotoxicity	Active 0.51	Inactive 0.59	Active 0.55	Active 0.5	Active 0.53	Active 0.53	Inactive 0.50	Active 0.52	Active 0.58	Active 0.5
Immunotoxicity	Inactive 0.98	Inactive 0.92	Inactive 0.91	Inactive 0.98	Inactive 0.95	Inactive 0.98	**Active** 0.79	**Active** 0.71	**Active** 0.99	Inactive 0.96
Carcinogenicity	Inactive 0.68	Inactive 0.71	Active 0.56	Inactive 0.67	Active 0.60	**Active** 0.81	Inactive 0.56	Active 0.63	Active 0.57	Inactive 0.62
Mutagenicity	Inactive 0.63	Inactive 0.65	Inactive 0.65	Inactive 0.58	Active 0.57	Inactive 0.62	Inactive 0.57	Active 0.51	Active 0.52	Active 0.55
Cytotoxicity	Inactive 0.7	Inactive 0.62	Inactive 0.68	Inactive 0.77	Inactive 0.70	Inactive 0.77	Inactive 0.64	Inactive 0.62	Inactive 0.68	Inactive 0.75
LD50(mg/Kg)	3000	3000	1000	3000	1000	1000	1000	1000	1000	3000
Toxicity class	5	5	4	5	4	4	4	4	4	5

Consensus score is indicated: < 0.3 not good prediction, 0.3–0.7 marginal prediction. > 7 strong predictions. Toxicity alerts with consensus scoring (CS) > 0.7 are highlighted in bold

In addition to the in silico predictions, in vivo toxicity was assessed using the hemolysis assay and the *G. mellonella* model as shown in Table 2. Most active compounds showed no induction of RBC damage or hemolysis at 200 μM, however UV296 and UV308 induced RBC hemolysis at lower concentrations. LD_50_ in *G. mellonella* classified most compounds as low or no toxic (Table 2) whereas in silico predicted LD_50_ was higher (Table 4). Remarkably, compound UV797 exhibited no toxicity alerts and low or no predicted toxicity in both in vitro and in vivo assays.

## Discussion

The spread of resistant parasites has driven the search for novel molecules for alternative malaria treatments. In this work, an in-house chemical library of novel heterocyclic compounds was designed based on biologically active scaffolds. In silico and in vitro analyses were performed to identify molecules with antimalarial activity and the potential mechanism of action. The potential antimalarial activity of the compounds was predicted for relevant target proteins from *P. falciparum* with a role in parasite survival. Two docking programs, Autodock Vina and Autodock 4, were used to improve predictions; although these platforms have been reported as comparable, the correlation between the data obtained may depend on the target; here, 5 out of 7 proteins showed better correlation. As suggested by others, Autodock 4 seems to have better binding predictions when the protein pockets are less polar and less charged, whereas Autodock Vina has better performance for docking of charged and polar pockets (Vieira and Sousa 2019). In this work, the combined in silico predictions identified 7 out of 10 most active compounds in vitro as top binders for one or several target proteins.

From the in vitro active compounds, 7 were predicted to bind *Pf*Prx1, and remarkably, the pyridyl-diaminopyrimido-diazepines (UV802, UV797, and UV801) showed submicromolar inhibition of parasite cultures. *Pf*Prx1 is one of the five peroxiredoxin proteins identified in *P. falciparum*, a family of thiol-dependent peroxidases. Peroxiredoxins are ubiquitous enzymes, particularly *Pf* Prx1, which is highly expressed in mature asexual stages of the parasite and considered a target for diagnosis and antimalarial action; however, the function of *Pf*Prx1 still needs to be further dilucidated as *Pf*Prx1 knock out (KO) parasites showed no effects on stress oxidation, parasite development, or morphology (Kimura et al. 2013). By contrast*,* 2-cys peroxiredoxin KOs induced hypersensitivity to heat stress (Kimura et al. 2013). It has been suggested that compounds inhibiting the parasite peroxiredoxins may be synergistic to other antimalarials by increasing the effects of induced oxidative stress (Kimura et al. 2013). Nevertheless, the predicted interaction of this protein class with the substituted diazepines described in this work should be further validated using functional and specific in vitro assays.

Four compounds (UV306, UV7, UV298, and UV186) were top ranked for *Pf*PMII, a protein which has gained relevance as an antimalarial target. *Pf*PMII is a protease involved in hemoglobin digestion and degradation into amino acids; as per literature, the loop from Gly291 to Pro297 is highly conserved and is involved in hemoglobin interaction, leading to protein cleavage (Liu et al. 2006). Previous studies have reported a series of piperazines and hydroxyethylamine with predicted action as plasmepsin inhibitors (Sharma et al. 2022); almost all candidates showed interactions with the experimentally reported crucial amino acids, i.e., Asp34 and Asp214 (Manhas et al. 2022). Here, all the inhibitory compounds with predicted *P*fPMII activity (two N-phenyl pyrazolines, quinazolyl- and quinolinyl- aminochalcones) had a dioxo substituent; however, for 3 of them, the predicted profile of toxicity showed a moderate level of immunotoxicity (cs > 0.7).

Two compounds (UV296 and UV 300) with in vitro inhibitory activity were top ranked for binding to heat shock protein, particularly the N-terminal ATP-binding domain of *P. falciparum* Hsp90. The Hsp90 is a chaperone with ATPase activity highly conserved across species. Four Hsp90s have been found expressed in *P. falciparum* in different cell compartments like the cytosol, endoplasmic reticulum, and mitochondria. The Hsp90s interact with a wide network of proteins (co-chaperons and clients proteins) and serve as modulators of several cellular processes including stress regulation, DNA repair, protein folding, and signaling pathways (Stofberg et al. 2021). These proteins are highly involved in protein quality control and may have a potential role in the erythrocyte stage development. Furthermore, inactivation of Hsp90 results on the degradation of client proteins; therefore, Hsp90s have been considered as a relevant antimalarial target (Stofberg et al. 2021). Although parasitic Hsp90 has a human homolog, it differs in some structure and functional aspects (Dutta et al. 2022); moreover, the increased ATPase activity in *P. falciparum* makes it more susceptible than the host’s cells (Stofberg et al. 2021). The most studied small molecules inhibiting Hps90 are ATP analogs mimicking the nucleotide structure and competitive binding to the ATP binding site. Compounds showing inhibition included the natural products ansamycin and resorcinol and synthetic compounds such as purine derivatives, benzamide, minopyri(mi)dines, and tricyclic imidazopyridines. Potent inhibitors like the 7-azandole derivative (IND31119) and an aminoalcohol-carbazole (N-CBZ IC_50_ 82 nM) selectively bind to the hydrophobic pocket from the base of the ATP binding site (Zininga and Shonhai 2019). Other families of inhibitors are the imidazopyridazines (Green et al. 2016) and the purine-based derivatives; from the latter, the compound PU-H71 showed to be active in vitro against *P. falciparum* (IC_50_ 110 nM) (Shahinas et al. 2013). In this study, compound UV296, a pyridyl-N-phenyl pyrazoline with a methoxy substitution, showed higher IC_50_ (IC_50_ 629 nM) than that reported for the PUH71.

Compound UV306 was also predicted to bind *Pf*HAD1. HAD protein family is linked to metabolic homeostasis from malaria parasites and the methylerythritol phosphate (MEP) pathway in the isoprenoid biosynthesis (Frasse et al. 2019). HAD proteins are mainly considered phosphatases distributed into three subfamilies (I, II, III). Most HAD, including HAD1, belong to the subfamily II, and loss of this protein induces resistance to fosmidomycin (a well-known inhibitor of the MEP pathway) and accumulation of sugar phosphate intermediates of this pathway (Guggisberg et al. 2014). These suggested a common, conserved, and essential role for HAD superfamily members and a potential focus of therapeutic strategies; however, studies exploring the role as drug targets are still underway. So far, studies by Mahapatra and Das (2020) have suggested that *Pf*HAD1 is one of the targets of genitianine, a phytochemical antimalarial chemically considered a pyranopyiridine (C_10_H_9_NO_2_) (Mahapatra and Das 2020). Here, we have predicted one pyridyl-amino-N-4-chlorophenyl pyrazoline and one quinazolylamino chalcone interacting with this protein, but the latter with discreet growth inhibition of cultures (IC_50_ 2.51 μM).

A multitarget interaction was predicted for inhibitory compound UV7 (IC_50_ 1.069 μM) with predicted binding to *Pf*PMII, *Pf*SpdS, *Pf*CDPK4, *Pf*Prx1, and *Pf*PNP. Although these predictions require further experimental validation, compounds with multiple targets are considered a suitable strategy to avoid or delay resistance, minimize drug combination, and easily adjust solubility issues. As the novel heterocyclics have been designed and synthetized using a multi-target hybrid approach, polypharmacology effects may be expected. These hybrid molecules have been designed by incorporating multiple chemical scaffolds of well-known and successful drugs to improve biological activity.

Consistently in this in-house library, the pyrimidines derivatives (diaminopyrimido-diazepines) and pyrazolines containing pyridine substituents showed the highest antimalarial activity suggesting the relevance of these ring systems. As stated by other authors, the antimalarial effects of pyrimidine have been reported in some cases linked to particular targets such as *P. falciparum* dihydrofolate reductase (*Pf*DHFR) and falcipain 2 (Dhameliya et al. 2023). Pyrimidine derivatives with antimalarial activity include morpholino-pyrimide-based distinctive pyrazole carboxamides, pyrazolylpyrimidine-based N-thioamide derivatives of piperazine, triazolopyrimidines, pyrimidine substituted 4-fluoroamodiaquines, and 2,4 diaminopyrimidine derivatives (Dhameliya et al. 2023). A recent review reported antimalarial activity (IC_50_ or EC_**50**_**)** of compounds with these scaffolds ranging from 0.0046 to 10 μM (Dhameliya et al. 2023). From the most potent compounds, the new triazolopyrimidines showed inhibition at the low nanomolar range against 3D7 strains (EC_50_ 0.0039–6.5 μM) and the *Plasmodium* dihydroorotate dehydrogenase (*Pf*DHODH) (IC_50_ 0.005–8 μM (Kokkonda et al. 2016). Tripathi et al. (2019) assessed hybrids of 4′-fluoro-amodiaquine and substituted pyrimidine showing better in vitro activity than CQ against *Pf*NF54 (IC_50_ 7.47 nM) and the resistant strain Dd2 (IC_50_ 4.69 nM) (Tripathi et al. 2019). The authors suggested that piperidine substitution in the pyrimidine ring resulted in forty-seven times higher activity than CQ. Here, pyridyl-diaminopyrimidine diazepines were the most potent antimalarials, this agrees with previous reports where the 1,4 diazepines have been recognized as peptidomimetic scaffolds and considered as pivotal for drug design (Meanwell and Walker 2008).

In addition to pyrimidines, other N-based heterocyclic structures have been widely reported to exhibit antimalarial activity. In this study, most active heterocyclic compounds had a pyridine substituent; this ring system is known by the broad range of pharmacology effects conferring improved biological activity, permeability, metabolic stability, and protein binding; indeed, 14% of approved drugs contain a pyridine ring (Ling et al. 2021). The pyridine ring system confers the ability of H bonding and hydrophobic noncovalent interactions leading to improved potency. Antimalarial activity of compounds bearing this scaffold ranges from 1.2 nM to 4.78 μM with diverse potential targets such as falcipain 2 (Dhameliya et al. 2023), cytochrome *bc*_*1*_ (Rodrigues et al. 2009), *Pf*DHFR (Bekhit et al. 2012), and plasmodial kinase (Le Manach et al. 2015). Several studies have reported the inhibitory activity of pyridines at the low micromolar or nanomolar level. Studies by Eagon et al. (2020) reported two pyrazolo[3,4-*b*] pyridines inhibiting *P. falciparum* with IC_50_ ranging from 0.070 to 0.4 μM and suggesting the Q_o_ binding site of cytochrome *bc*_1_ as a potential target (Eagon et al. 2020). Other studies in vitro have reported the antimalarial activity of pyrazole pyridines against *P. falciparum* (K1 and NF54) and *P. berghei* strains; for instance, Manach et al. (2015) described a novel series of pyrazolopyridines with IC_50_ lower than 50 nM against *P. falciparum* (Le Manach et al. 2015).

Novel pyridines with antimalarial activity include a wide variety of synthetic pyrazolopyridine derivatives and benzenesulfonamide hybrids (Silva et al. 2016), a novel series of acyl hydrazone-based hybrids of 1,4- dihydropyridine (Kumar et al. 2017; Ravindar et al. 2023) and highly active fosmidomycin derivatives containing the pyridine ring and showing potent inhibition of plasmodial 1-deoxy-d-xylulose 5-phosphate reductoisomerase (DXR) with 11-fold higher activity than fosmidomycin (Xue et al. 2013). In vitro testing of novel 4 (2 amino-1 hydroxyethyl)pyridines against *Pf*W2 and *Pf*3D7 strains showed IC_50_s from 3.5 to 10 nM and from 17.7 to 56.7 nM, respectively, being more potent than CQ and mefloquine (Bentzinger et al. 2020). Younis et al. found that methoxypyridyl substitution of 3,5-diaryl-2-aminopyridines confers potent activity with IC_50_ values of 25.0 nM and 28.0 nM against K1 and NF54 strains (Rathod et al. 2022). Recently, the imidazopyridine scaffold has been recognized to have tremendous biological potential for its antimalarial kinase inhibition (Le Manach et al. 2015). From the above evidence, pyridine-based compounds have great potential for the design and development of novel antimalarial drugs, particularly hydrogen-bond interactions between the pyridine nitrogen and the cysteine of the parasite target proteins seem to be key for inhibition (Ling et al. 2021).

Other pharmacophoric moiety exhibiting a great and diverse biological activity is the substituted pyrazoline ring system. Antiparasitic activity of trisubstituted pyrazoline derivatives against several species of *Plasmodium* has been reported. Trisubstituted pyrazoline derivatives have been reported active against CQ-sensitive (MRC-2) (IC_50_ 0.022 μM) and CQ-resistant (RKL-9) (IC_50_ 0.192 μM) (Mishra et al. 2017). Acharya et al. (2010) reported potent in vitro inhibition of 1,3,5 trisubstituted pyrazolines containing further methoxy and hydrogen substitutions (CQ-resistant IC_50_ 0.0425 μM and CQ-sensitive IC_50_ 0.0265 μM strains of *P. falciparum)* (Acharya et al. 2010); the antimalarial activity of trisubstituted pyrazoles was correlated with β-hematin inhibition. Likewise, a series of oxazoline-pyrazoline hybrids exhibited antimalarial effects against CQ-sensitive *Pf*3D7 (IC_50_ 0.322 μg/mL) and CQ-resistant *Pf*RKL9 (IC_50_ 0.192 μM) (Pandey et al. 2016). A recent work by Ekawati et al. (2022) showed that 3-styryl-2-pyrazolines with N-acetyl and N-phenyl substitutions pyrazoline had antimalarial activity (0.101–5.695 μM); particularly, the presence of methoxy groups on the N-phenyl pyrazolines improved antimalarial effect (0.101–0.177 μM); by contrast, N-acetyl pyrazolines containing up to two methoxy groups showed less potency (2.156–5.695 μM). Docking analysis of N-phenyl pyrazolines revealed potential binding to *Pf*DHR and similar interactions with the control ligand; nevertheless, toxicity of the compounds was detected (Ekawati et al. 2022). In agreement with Ekawati et al. (2022), we found that pyridylamino substituted N-phenyl pyrazolines having one methoxy group were more active, whereas the presence of methoxy group on pyridylamino substituted N-acetyl pyrazolines decreased activity. Nevertheless, trimethoxy substituents decreased antimalarial activity in both pyrazoline types and particularly for the pyridylamino substituted N-acetyl pyrazolines. By contrast with Ekawati et al. (2022), we used different protein targets for docking, the most potent N-phenyl pyrazoline was top ranked for binding to *Pf*Hsp90; however, the active pyridylamino-N-acetyl pyrazoline showed no relevant binding to the selected protein targets suggesting an alternative mechanism of action. As suggested in the literature, pyrazoline derivatives interfere with β-hematin formation and haem crystallization, indicating that these active heterocyclics should be assessed to test the effects on this parasite process. Although in this work some toxicity alerts were predicted for the pyridylamino-N-acetyl and N-phenyl substituted pyrazolines, the most active pyrazolines showed low toxicity risk when compared to the predicted for the previously reported N-acetyl and N-phenyl substituted styryl pyrazolines (Ekawati et al. 2022). In our study, toxicity was validated in vivo using *G. mellonella*, and this model has shown strong data correlation when compared to mammalian models and provides a more accurate representation of in vivo toxicity than in vitro cell-based assays (Allegra et al. 2018; Piatek et al. 2021).

## Conclusions

Using structure-based virtual screening combined with in vitro assays, this study has identified 8 hits from three different chemical series with a predicted mechanism of action. From the results, compounds UV802, UV797, UV286, UV801, UV296, UV292, UV306, and UV297 showed satisfactory ADMET properties and inhibition of parasite cultures (IC_50_ < 1 μM). Diaminopyrimido-diazepines UV802, UV797, and UV801 showed the highest activity and in silico predicted binding with *Pf*Prx1 suggesting this protein as a potential target. These compounds contained no toxicophore and complied with the LRo5 and drug-likeness parameters.

Particularly, UV802 was the most potent and drug-like nontoxic compound with in vitro antimalarial activity at the low submicromolar range and presumptively interacting with *Pf*Prx1. This hit could be improved by the synthesis of novel analogues to further progress into the drug discovery pipeline. We have also described the pharmacophore potentially inhibiting the *Pf*Prx1; nevertheless, this postulated mechanism of action and its specific effects on parasite erythrocytic stages or CQ-resistant strains require further experimental validation.

### Supplementary Information

Below is the link to the electronic supplementary material.

Supplementary file1 (DOCX 191 KB)

Supplementary file2 (DOCX 184 KB)

## Data Availability

Not applicable.
