# Supplementary material for: In silico prediction and in vitro assessment of novel heterocyclics with antimalarial activity
Source: Parasitol Res. 2023 Dec 29;123(1):75. doi: 10.1007/s00436-023-08089-7 (PMC10754745; doi:10.1007/s00436-023-08089-7)

**Supplementary Fig. 1** Autodock Vina and Autodock 4 correlation. Scatter of rankings by target protein.


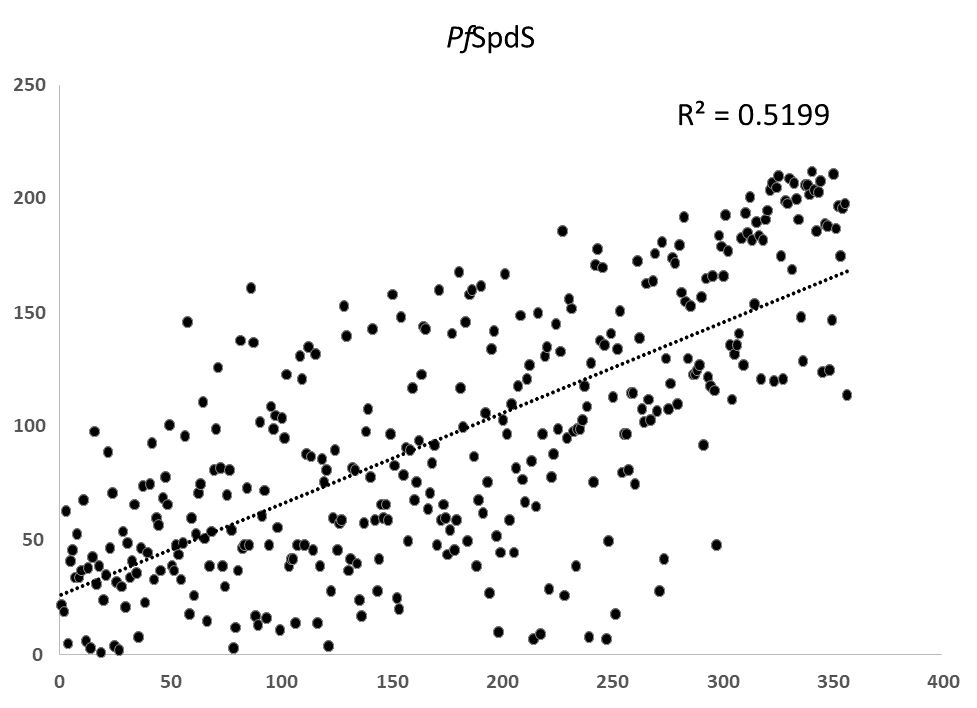

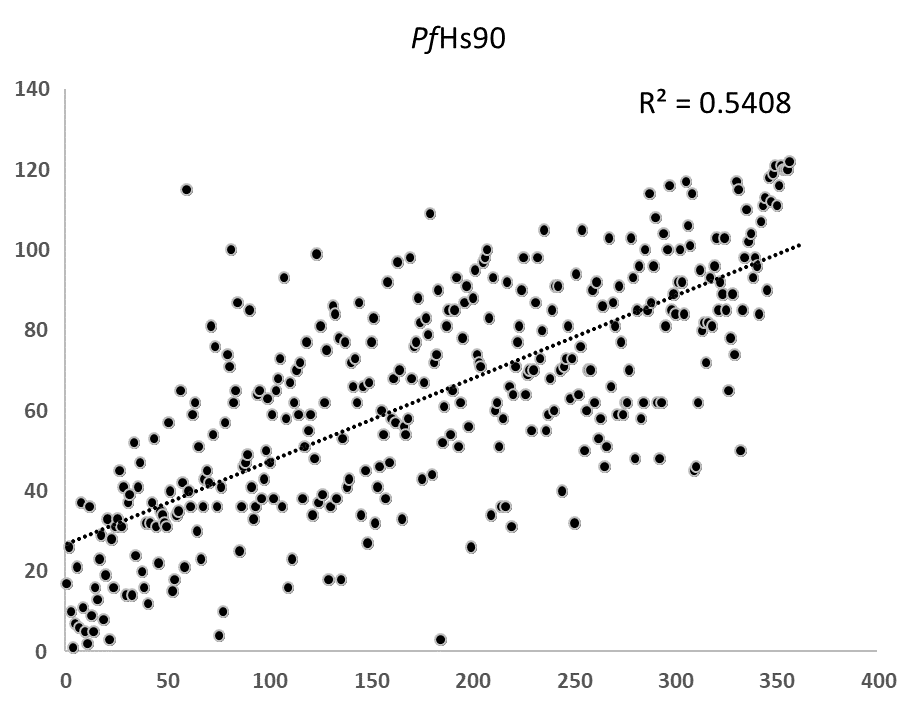

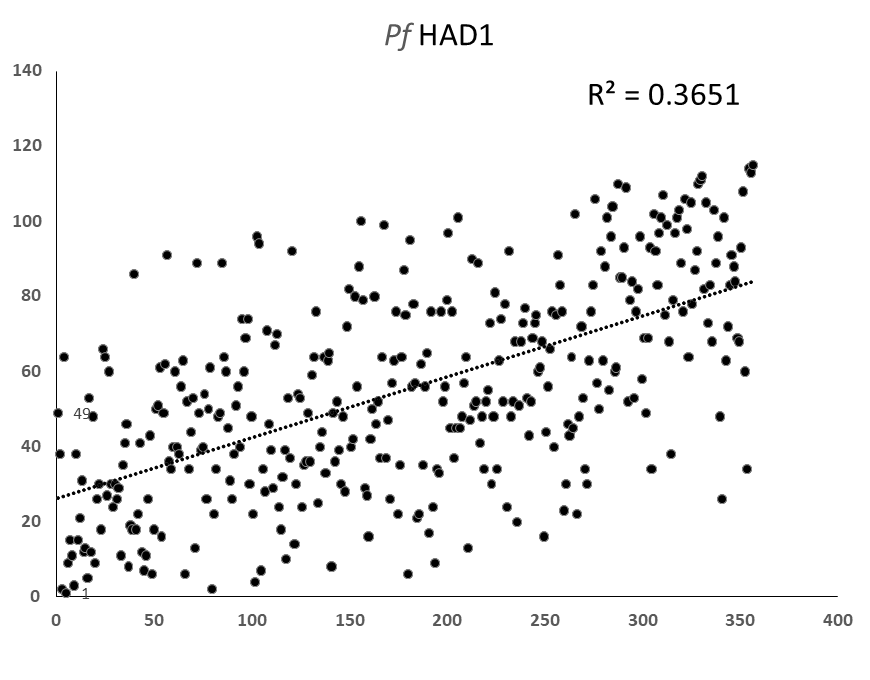

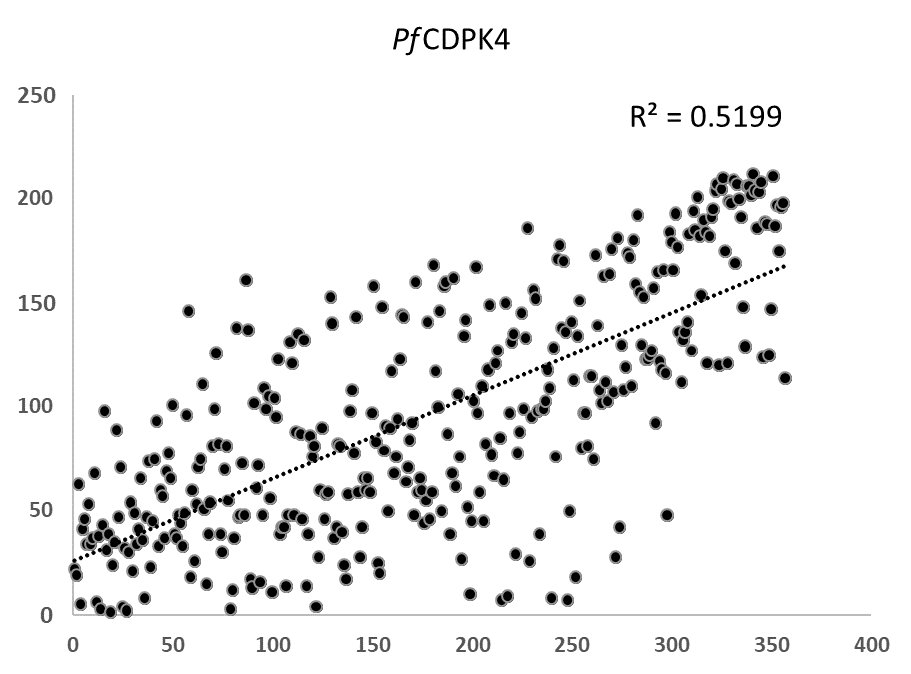

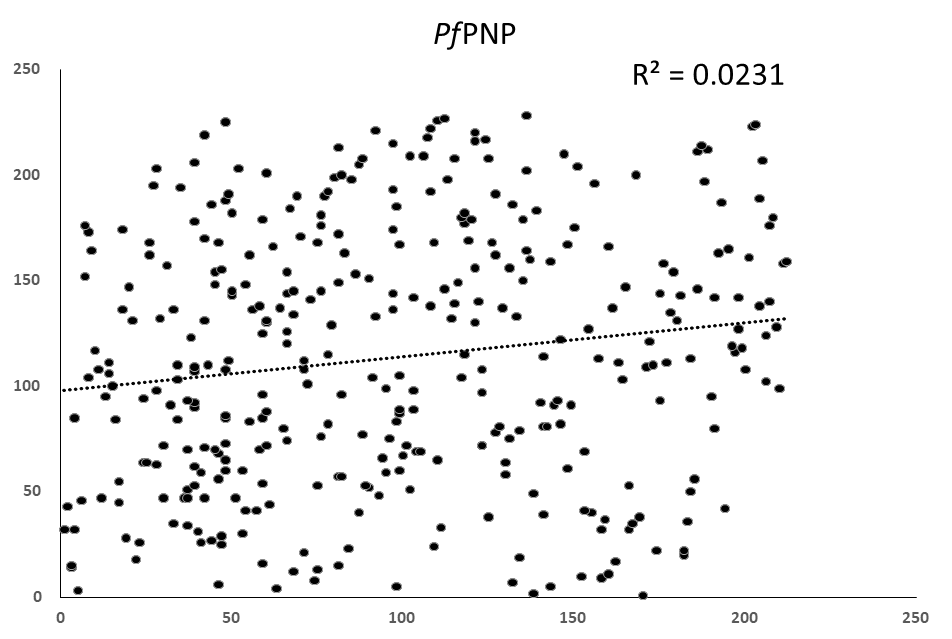

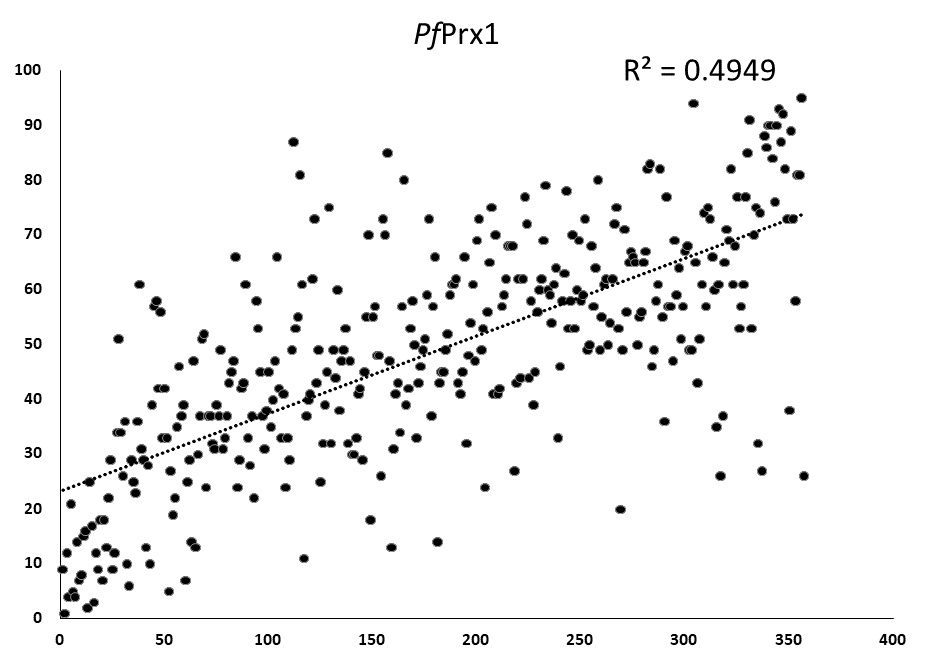


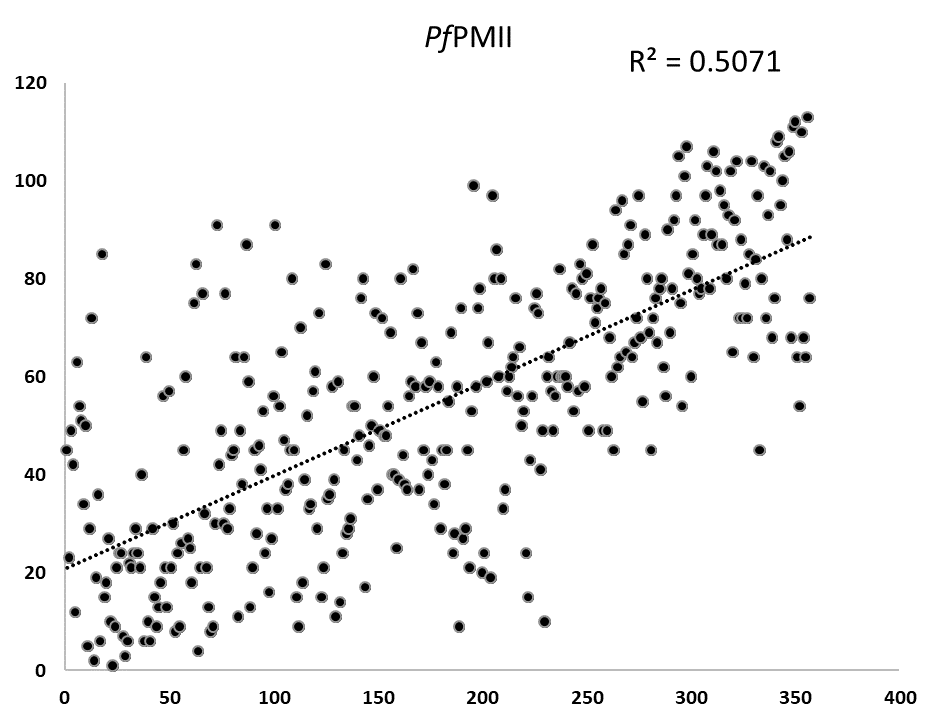

Supplement: Supplementary file 1 — Supplementary file1 (DOCX 191 KB) [file 436_2023_8089_MOESM1_ESM.docx]
