# Supplementary material for: In silico prediction and in vitro assessment of novel heterocyclics with antimalarial activity
Source: Parasitol Res. 2023 Dec 29;123(1):75. doi: 10.1007/s00436-023-08089-7 (PMC10754745; doi:10.1007/s00436-023-08089-7)
Supplement: Supplementary file 2 — Supplementary file2 (DOCX 184 KB) [file 436_2023_8089_MOESM2_ESM.docx]

Supplementary Table 1. Structure of *in silico* top 10 ranked heterocyclic compounds (n=37)

| **Compd**  **(UV)** | **Structure** | **Chemical name** | **Compd**  **(UV)** | **Structure** | **Chemical name** |
| --- | --- | --- | --- | --- | --- |
| 5 |  | Quinolinyl-amino  chalcone | 183 |  | Quinazolyl-amino  chalcone |
| 7 |  | Quinolinyl-amino  chalcone | 186 |  | Quinazolyl-amino  chalcone. |
| 15 |  | Quinolinyl-amino-N-acetyl-pyrazoline | 187 |  | Quinazolyl-amino  chalcone |
| 17 |  | Quinolinyl-amino-N-acetyl-pyrazoline | 200 |  | N-(4-chlorobenzyl)  indolyl-N-acetyl-pyrazoline |
| 21 |  | Quinolinyl-amino-N-formyl-pyrazoline | 220 |  | Quinoline  chalcone |
| 23 |  | Quinolinyl-amino-N-formyl-pyrazoline | 226 |  | Quinoline  chalcone |
| 96 |  | Thiazolyl-indazole | 231 |  | Quinoline  chalcone |
| 114 |  | N'-isonicotinoyl-sulfonyl-hydrazide-chalcone | 232 |  | Quinoline  chalcone |

| **Compd**  **(UV)** | **Structure** | **Chemical name** | **Compd**  **(UV)** | **Structure** | **Chemical name** |
| --- | --- | --- | --- | --- | --- |
| 245 |  | Quinolinyl-amino  chalcone | 526 |  | Triazinyl-  amino  chalcone |
| 247 |  | Quinolinyl-amino  chalcone | 619 |  | Benzoindazole |
| 296 |  | Pyridyl-amino  pyrazoline | 624 |  | Benzoindazole |
| 298 |  | Pyridyl-amino-N-phenyl-pyrazoline | 629 |  | Triazinyl-aminoamido-sulfonyl-cetophenone |
| 300 |  | Pyridyl-amino-N-phenyl-pyrazoline | 645 |  | Triazinyl-amino-chalcone |
| 306 |  | Pyridyl-amino-N-4-chlorophenyl-pyrazoline | 646 |  | Triazinyl-amino-chalcone |
| 497 |  | Quinazolyl  amino-N-formyl-pyrazoline | 697 |  | Quinoline  chalcone |
| 506 |  | Triazinyl-amino  chalcone | 704 |  | Quinoline  chalcone |
| **Compd**  **(UV)** | **Structure** | **Chemical name** | **Compd**  **(UV)** | **Structure** | **Chemical name** |
| 712 |  | Pyridyl-amino-pyrimido-diazepine | 801 |  | Pyridyl-amino-  pyrimido-  diazepine |
| 774 |  | Pyrimidyl-  chalcone | 802 |  | Pyridyl-amino-pyrimido-diazepina |
| 797 |  | Pyridyl-amino-pyrimido-diazepine |  |  |  |

Compd: compound.
